# CircRNAs: biogenesis, functions, and role in drug-resistant Tumours

**DOI:** 10.1186/s12943-020-01231-4

**Published:** 2020-08-05

**Authors:** Shuo Ma, Shan Kong, Feng Wang, Shaoqing Ju

**Affiliations:** 1 Department of Laboratory Medicine, Affiliated Hospital of Nantong University, NO.20, Xisi Road, Nantong, 226001 Jiangsu China; 2 Research Center of Clinical Medicine, Affiliated Hospital of Nantong University, NO.20, Xisi Road, Nantong, 226001 Jiangsu China; 3 School of Public Health, Nantong University, NO. 9, Seyuan Road, Nantong, 226019 Jiangsu China

**Keywords:** Circular RNAs (CircRNAs), Tumor resistance, Targeted treatment, Self-circularize, Functions

## Abstract

Targeted treatment, which can specifically kill tumour cells without affecting normal cells, is a new approach for tumour therapy. However, tumour cells tend to acquire resistance to targeted drugs during treatment. Circular RNAs (circRNAs) are single-stranded RNA molecules with unique structures and important functions. With the development of RNA sequencing technology, circRNAs have been found to be widespread in tumour-resistant cells and to play important regulatory roles. In this review, we present the latest advances in circRNA research and summarize the various mechanisms underlying their regulation. Moreover, we review the role of circRNAs in the chemotherapeutic resistance of tumours and explore the clinical value of circRNA regulation in treating tumour resistance.

## Background

Circular RNAs (circRNAs) are newly recognized non-coding RNAs that are considered small endogenous RNAs with a wide distribution, considerable variety, and multiple regulatory functions [1]. In 1976, Sanger et al. discovered the first circRNA in viroids [2]. Since then, tens of thousands of circRNAs have been identified in multiple cell lines and species [3–5], including fungi, protozoa, plants, worms, fish, insects, mice and humans [6–8]. CircRNAs are abundant—approximately one-eighth of the genes in the human transcriptome can produce detectable circRNAs, and the expression levels of these circRNAs are more than ten times those of the corresponding linear mRNAs [9, 10]. Additionally, circRNAs are more stable than linear RNAs because of their covalent closed-loop structure and lack of free terminal ends, which confers resistance to degradation by ribonuclease R (RNase R) [11]. Moreover, circRNAs are evolutionarily conserved. Approximately 15,000 human circRNA sequences can be detected in mouse or rat genomes [5, 12]. CircRNAs can also be used to classify and identify different tumour types due to their advantage of cell type-, tissue-, and developmental stage- specific expression and because different subtypes of circRNAs can be produced [13–16]. Considering the above observations, we believe that circRNAs have great research potential. As research has progressed, various biological functions of circRNAs have been revealed. CircRNAs can act as “sponges” for microRNAs (miRNAs) and affect the function of miRNA target genes [17]. In addition, circRNAs can bind to specific RNA binding proteins (RBPs), thereby affecting the function of the parental genes [18–20]. Intriguingly, accumulating evidence shows that circRNAs can encode proteins/peptides that are involved in tumour pathogenesis and progression [21–23]. The unique properties and biological functions of circRNAs demonstrate the importance of circRNAs in tumorigenesis, proliferation, metastasis, invasion, and drug resistance, which also suggests the possibility that circRNAs can be used as biomarkers and tumour therapeutic targets [24–26].

Tumour treatment remains a serious medical problem worldwide. Despite clinical advances, chemotherapy and radiotherapy are still the preferred methods for tumour treatment. The development of drug resistance means that tumour cells can evade the effects of antitumour drugs with different structures and functions, and drug resistance has proven an important obstacle to tumour treatment [27, 28]. Due to the evolution of drug resistance, a considerable number of cancer patients experience local recurrence and distant metastasis, which may lead to poor prognosis and higher tumour mortality. Although extensive studies have been conducted, the mechanisms of and responses to drug resistance in tumours remain unclear. Several studies have shown that miRNAs and long non-coding RNAs (lncRNAs) are associated with chemotherapeutic resistance [29, 30]. However, information on the involvement of circRNAs in drug resistance and the underlying regulatory mechanisms is scarce.

In this review, we provide an overview of the biosynthesis and clinically significant of circRNAs and describe the differential expression of circRNAs in drug-resistant tumours. We emphasize potential regulatory mechanisms of circRNAs to provide a basis for clinical treatment.

## Biogenesis and characteristics of circRNAs

### Biological origin of circRNAs

**Fig. 1:**
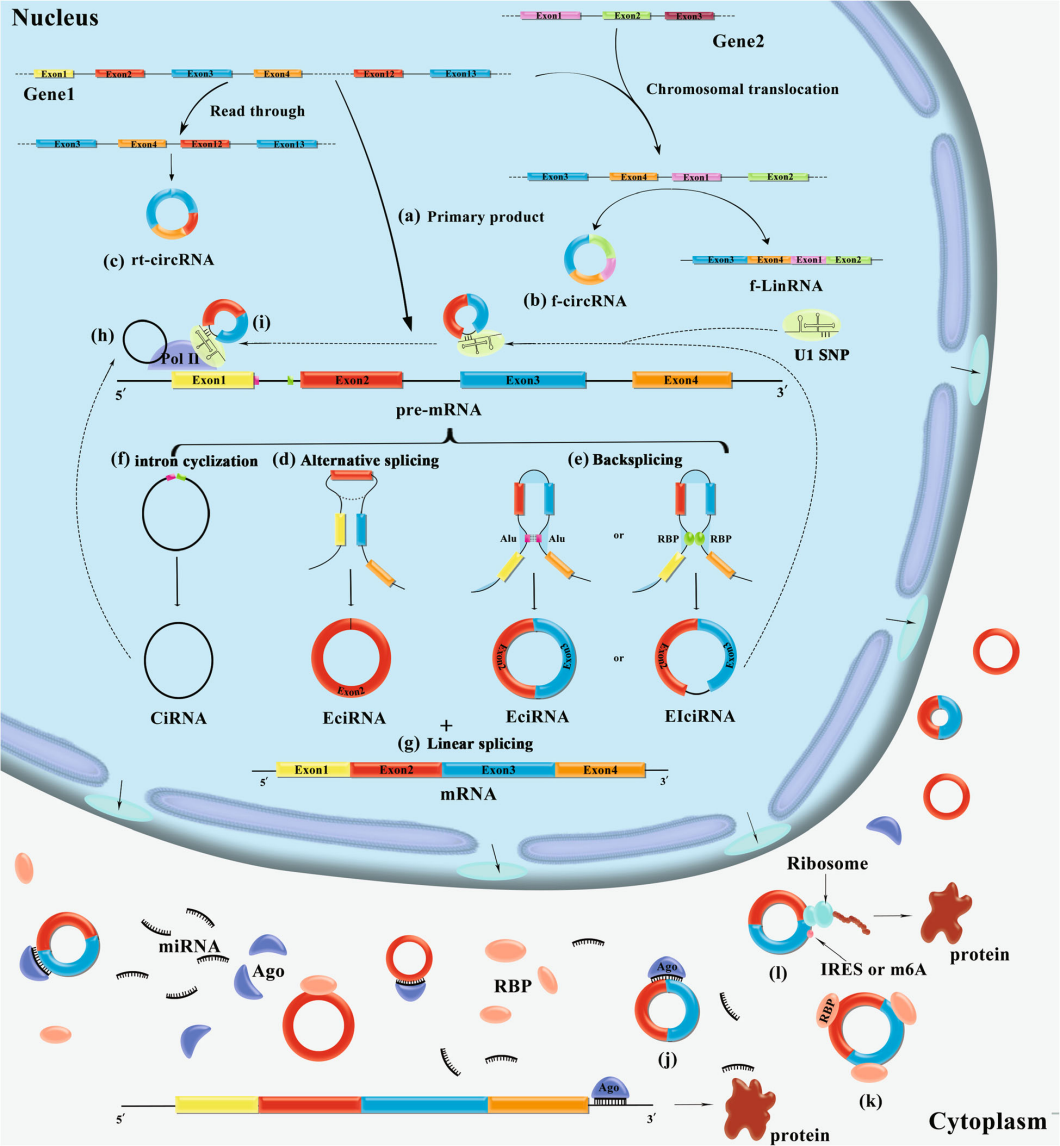
Biogenesis and function of circRNAs. **a**. The main product of gene transcription, pre-mRNA. **b**. In vivo, chromosomal translocations and other reasons may cause CircRNAs to be produced by exons of different genes, which are called fusion CircRNAs(f-circRNAs). **c**. Two adjacent gene exons on the same DNA chain can form circRNAs, read through circRNA (rt-circRNAs). **d**. During pre-mRNA transcription to produce a lariat structure containing exons, which is then spliced internally to release introns and form EciRNAs composed of exons. **e**. the tail end of the downstream 3′ splicing donor site in the exon binds to the upstream 5′ splicing receptor site, resulting in base pairing of the donor and receptor sites, which mediates exon circularization to form circRNAs or EciRNA. It is regulated by Alu, RBPs, and parental gene exons. **f**. The interconnections of introns cause the formation of CiRNAs after the lariat structure undergoes internal reverse splicing. **g**. Liner mRNA: single-stranded ribonucleic acids are carrying genetic information. **h**. Interaction between CiRNA and Pol II promotes parental gene transcription. **i**. EIciRNAs interact with U1-SNP and Pol II to enhance gene expression. **j**. CircRNAs can affect the occurrence and development of diseases as competitive binding miRNA of ceRNA. **k**. CircRNAs act as RBPs scaffold sponge to regulate variable splicing of transcripts, transcription of parent genes, and post-transcriptional translation. **l**. CircRNAs can be modified with IRES sequence and m^6^A to facilitate the translation of circRNAs

CircRNAs are divided into three categories according to their source: exonic circRNAs (EciRNAs), exon-intron circRNAs (EIciRNAs), and intronic circRNAs (CiRNAs) [1, 31, 32]. Most circRNAs are formed by exon skipping during pre-messenger RNA (pre-mRNA) transcription to produce a lariat structure containing exons, which is then spliced internally to release introns and form EciRNAs composed of exons [33–35]. Alternatively, the tail end of the downstream 3′ splicing donor site in the exon binds to the upstream 5′ splicing receptor site, resulting in base pairing of the donor and receptor sites, which mediates exon circularization to form circRNAs. Generally, circRNAs are produced from a single exon, but circRNAs containing several exons can also be formed [3]. The exons that form circRNAs are mainly contained in the same gene. Further study indicated the generation of circRNAs produced by exons from different genes due to chromosomal translocations and other reasons in vivo; these circRNAs are called fusion circRNAs (f-circRNAs), and most are oncogenes [36]. In addition, read-through circRNAs (rt-circRNAs) composed of two adjacent gene exons on the same DNA strand, were found by exon sequencing [37]. Rt-circRNAs accounted for only a small fraction (2.5% on average) of all circRNAs in each sample. The expression of rt-circRNAs is lower than that of total circRNAs, and the formation of rt-circRNAs is related to read-through of RNA polymerase II (RNA Pol II) at the gene locus [37]. During the formation of circRNAs, if introns between exons are retained, circular transcripts form EIciRNAs composed of both exons and introns [9, 38]. The interconnections of introns cause the formation of CiRNAs after the lariat structure undergoes internal reverse splicing [32]. The mechanisms of circRNA biogenesis are depicted in Fig. 1.

CircRNA biogenesis is regulated by many factors. Rei et al. found that mammalian-wide interspersed repeats (MIRs) mediate the biogenesis of circRNAs. Knockout of upstream or downstream MIRs in human and mouse genomes significantly inhibited the production of CDR1as [39]. N^6^-methyladenosine (m^6^A) has been demonstrated to affect the production of circRNAs [40]. From the pachytene stage to the round cell stage of spermatogenesis after meiosis, a large number of circRNAs with an extended m^6^A-modified open reading frame (ORF) are produced. Anti-m^6^A RNA binding protein immunoprecipitation (RIP)-seq data showed that as spermatogenesis progresses, the number of m^6^A-carrying circRNAs increases, proving that m^6^A can mediate the biogenesis of circRNAs. Di et al. confirmed that some circRNA-specific m^6^A loci may be related to the production of circRNAs. The authors found that circZNF609 contains a circRNA-specific m^6^A locus and that when the specific site is mutated, the production of circZNF609 is significantly inhibited [41]. In addition, base pairing between Alu elements and dimerization of RBPs in introns play essential regulatory roles in the formation of circRNAs alternative splicing [33, 42]. For example, the protein HQK is encoded by quaking (QKI) [43], fused in sarcoma (FUS) [44], and serine/arginine-rich splicing factor 3 (SRSF3) [45]. Via the Mini gene reporting system constructed in Drosophila cells, Liang et al. found that the trans splicing factor SR protein and heterogeneous nuclear ribonucleoproteins (hnRNPs) could interact with intron repeat sequences. In addition to the genes related to transcription termination, SR and hnRNPs can significantly increase the abundance of circRNAs [46]. A study showed that ATP-dependent RNA helicase A (DHX9) can promote the unwinding of double-stranded RNA structures and that its knockout can significantly increase the number and types of circRNAs [47]. The protein product of the interleukin enhancer-binding factor 3 (ILF3) gene, NF90/NF110, promotes the formation of circRNAs and participates in host antiviral mechanisms by stabilizing CiRNA pairs [48]. Studies have also shown that the parental genes of circRNAs with high abundance and constitutive detection have longer and larger introns on both sides of the circRNA [16] and contain greater numbers of repeating elements [37] than parental genes of circRNAs with low abundance and limited expression. However, compared with the parental genes of circRNAs, parental genes of rt-circRNAs have longer introns and more repeating elements in their flanking sequences [37]. These elements draw the two splice sites closer together and facilitate reverse splicing [7]. Josh et al. found that parental genes of circRNAs tended to form multiple circular isomers and that the number of isomers formed was proportional to the number of exons in the parental gene [37]. However, the expression levels of circRNAs and the abundance of mRNA produced by the parental gene were not significantly related [4, 49]. In addition, circRNAs are downregulated in most tumours and are negatively correlated with cell proliferation [37]. The substantial accumulation of circRNAs in ageing neural tissue can also be explained by this observation [50].

### Characteristics of circRNAs

**Fig. 2:**
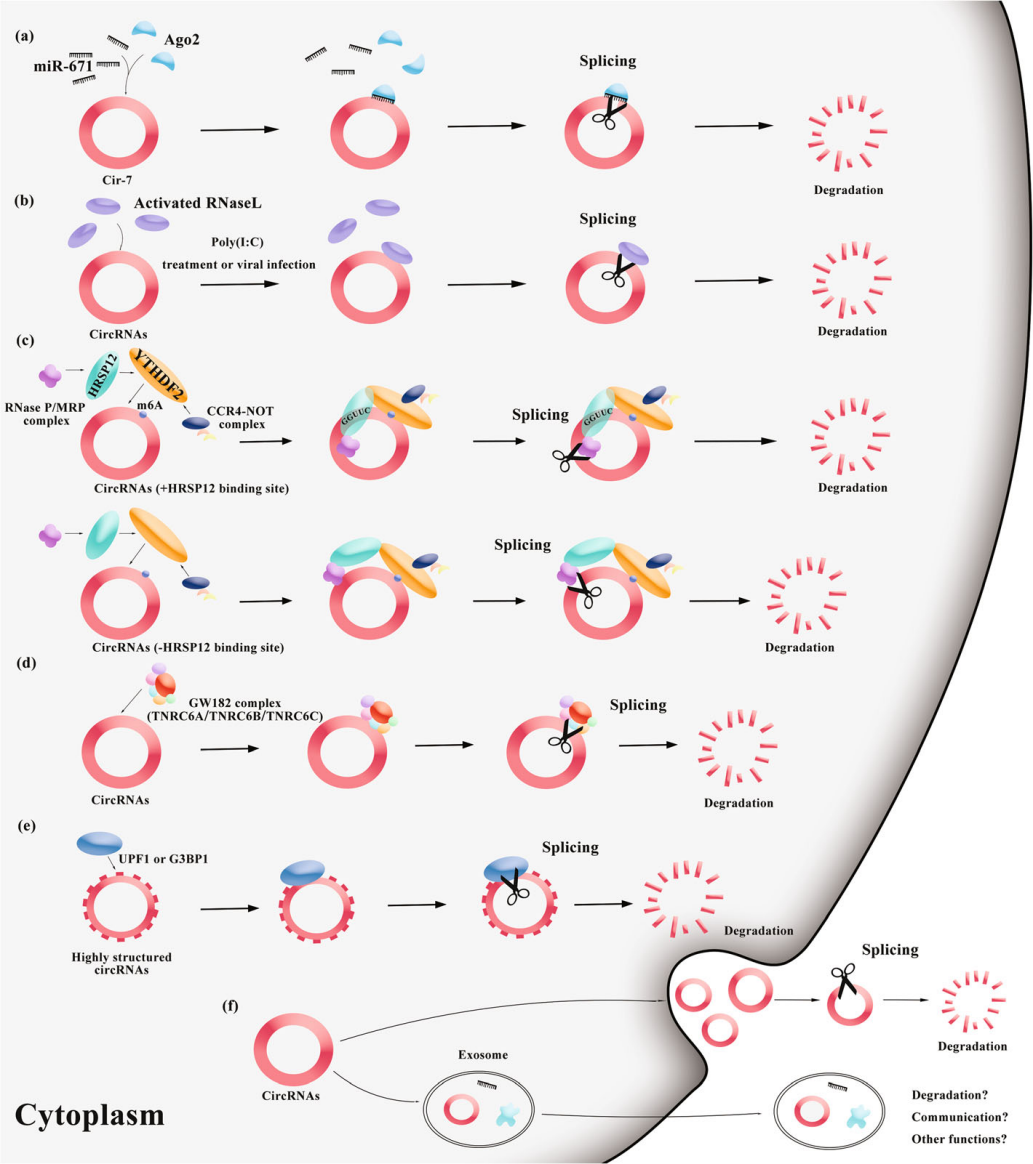
CircRNAs self-circularize mechanisms. **a.** MiR-671 binds to a highly complementary miRNA binding site in ciRS-7, thereby triggering the cleavage of AGO2. **b.** When cells are stimulated or infected by viruses, RNase L activates and degrades circRNAs molecules. **c.** CircRNAs with m^6^A sequences have two ways of self-renewing. **d.** The GW182 gene in drosophila cells and the human homologous genes TNRC6A, TNRC6B, and TNRC6C mediate the apoptosis of circRNAs. **e.** The regulation of UPF1 or G3BP1 can degrade CircRNAs with complex structures. **f.** CircRNAs are removed from the cytoplasm by exosomes or direct release into the extracellular space

Unlike linear RNAs, circRNAs are single-stranded, covalently closed circular transcripts without 5 ’caps and 3’ tails [9]. Thus, circRNAs are considered to be more stable than linear RNAs. In 2006, Hitoshi et al. demonstrated that circRNAs are not easily degraded by RNA exonucleases [51]. In 2015, Yehoshua et al. found that most circRNAs have longer half-life than their linear counterparts [52], especially in non-dividing cells [53]. However, because circRNAs are relatively stable, they can accumulate in cells with slower rates of division and thus affect cell functions. To date, five pathways of circRNA self- circularization that manage this limitation have been found (Fig. 2).

(1) MiR-671 binds to a highly complementary miRNA binding site in ciRS-7 (also called CDR1as), thereby triggering the cleavage of Argonaute 2 (Ago2) [54, 55]. Argonaute proteins play a role in RNA interference (RNAi) [56]. This cleavage can be enhanced by miR-7, which recruits the miR-671 silencing complex to ciRS-7 or retains it there through an undefined mechanism [55].

(2) CircRNAs containing m^6^A sequences can interact with YTH domain-containing family protein 2 (YTHDF2) (an m^6^A reader) [57] and possibly self-circularize via two mechanisms. CircRNAs that contain binding sites for heat-responsive protein 12 (HRSP12) (an adaptor protein) are preferentially degraded via ribonuclease P (Rnase P)/MRP-mediated endoribonucleolytic pathways [58], which are coupled to the CCR4-NOT complex-mediated deadenylation pathway via cooperative binding of HRSP12 and YTHDF2 to circRNAs containing m^6^A [59]. CircRNAs containing m^6^A lack HRSP12 binding sites, and YTHDF2 may still interact with HRSP12 and induce decay, albeit with low efficiency [59]. The HRSP12 binding site and the Rnase P/MRP cleavage site are located upstream and downstream of the YTHDF2 recognition site, respectively [59].

(3) CircRNAs tend to form a 16–26 bp small ring structure, which can bind and inhibit double-stranded DNA in the RNA-activated protein kinase (PKR) gene [60]. When cells are stimulated or infected with viruses, ribonuclease L (Rnase L) is activated and degrades circRNA molecules; subsequently, PKR is released and activates the downstream antiviral mechanism [60].

(4) GW182 mediates the degradation of circRNAs in Drosophila cells, as determined in studies based on RNAi library screening. Interference with GW182 minimally affects the abundance of nuclear circRNAs but, conversely, significantly decreases the abundance of cytoplasmic circRNAs. Overexpression of GW182 can lead to a decrease in the abundance of related circRNAs [61]. Moreover, circRNAs can be enriched significantly after interference with the three human homologues of the GW182 gene (TNRC6A, TNRC6B, and TNRC6C), indicating that TNRC6A/B/C are involved in the degradation of circRNAs [61].

(5) UPF1 and G3BP1 mediate the structure-mediated RNA decay (SRD) of mRNA. The abundances of mRNAs with a complex 3′ untranslated region (3’UTR) structure is less altered after UPF1 knockdown than those of mRNAs with less complex 3’UTR structures. Subsequently, this mechanism was confirmed to apply to circRNAs. The authors knocked down UPF1 and G3BP1 in DLD cells containing circRNAs with complex structures and analysed the abundance trends. The abundances of circRNA molecules with more complex structures changed less than those of circRNA molecules with less complex structures after knocking down UPF1 and G3BP1. Thus, circRNA molecules with complex structures may be regulated by the SRD mechanism [62].

In addition, studies have shown that circRNAs may be secreted from cells via exosomes [63, 64]; most of these circRNAs mediate inter-cellular communication through exosomes [65, 66]. However, whether this event is related to the self-regulation of circRNAs and whether the degradation of these circRNAs affects their cellular function are unclear.

### Nuclear and cytoplasmic transport of circRNAs

Most circRNAs are exported to the cytoplasm after formation, acting as miRNA sponges, binding with RBPs, or encoding proteins [67–69]. Huang et al. found that Hel25E is a vital regulator of post-transcriptional nucleation of circRNAs in Drosophila. Both of the Hel25E homologues, URH49 (DDX39A) and UAP56 (DDX39B), can mediate the nuclear export of circRNAs [70]. Interestingly, UAP56 and Hel25E are responsible mainly for the nuclear export of long circRNAs, whereas URH49 is responsible mainly for the nuclear export of short circRNAs [70]. Experimental results showed that circRNAs of different lengths exhibit different protein recognition characteristics due to point mutations in four amino acids located in the middle of the protein sequence [70]. In addition, circRNAs can rely on m^6^A for nuclear export [71]. m^6^A-modified circNSUN2 can bind YTH domain-containing family protein 1 (YTHDC1) and promote its nuclear export. YTHDC1 is a reader of RNA m^6^A [72]. CircNSUN2 interacts with YTHDC1 and can be enriched by antibodies against m^6^A. This modification mediates its interaction with YTHDC1. After interference with YTHDC1, the subcellular distribution of circNSUN2 changed, and the abundance of nuclear circNSUN2 increased [72]. This phenomenon was confirmed with fluorescence in situ hybridization (FISH) positioning analysis [71].

## CircRNA functions

With the development of research, the biological functions of circRNAs have been widely revealed [32, 73] (Fig. 1 and Table 1). 
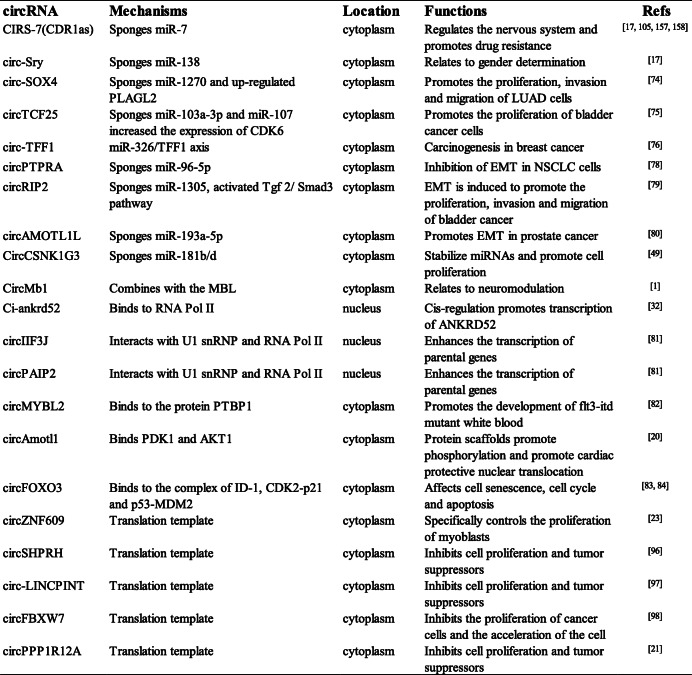

**Table 1:** The mechanisms and biological functions of main circRNAs

circRNA	Mechanisms	Location	Functions	Refs
CIRS-7(CDR1as)	Sponges miR-7	cytoplasm	Regulates the nervous system and promotes drug resistance	[17, 105, 157, 158]
circ-Sry	Sponges miR-138	cytoplasm	Relates to gender determination	[17]
circ-SOX4	Sponges miR-1270 and up-regulated PLAGL2	cytoplasm	Promotes the proliferation, invasion and migration of LUAD cells	[74]
circTCF25	Sponges miR-103a-3p and miR-107 increased the expression of CDK6	cytoplasm	Promotes the proliferation of bladder cancer cells	[75]
circ-TFF1	miR-326/TFF1 axis	cytoplasm	Carcinogenesis in breast cancer	[76]
circPTPRA	Sponges miR-96-5p	cytoplasm	Inhibition of EMT in NSCLC cells	[78]
circRIP2	Sponges miR-1305, activated Tgf 2/ Smad3 pathway	cytoplasm	EMT is induced to promote the proliferation, invasion and migration of bladder cancer	[79]
circAMOTL1L	Sponges miR-193a-5p	cytoplasm	Promotes EMT in prostate cancer cells	[80]
CircCSNK1G3	Sponges miR-181b/d	cytoplasm	Stabilize miRNAs and promote cell proliferation	[49]
CircMb1	Combines with the MBL	cytoplasm	Relates to neuromodulation	[1]
Ci-ankrd52	Binds to RNA Pol II	nucleus	Cis-regulation promotes transcription of ANKRD52	[32]
circIIF3J	Interacts with U1 snRNP and RNA Pol II	nucleus	Enhances the transcription of parental genes	[81]
circPAIP2	Interacts with U1 snRNP and RNA Pol II	nucleus	Enhances the transcription of parental genes	[81]
circMYBL2	Binds to the protein PTBP1	cytoplasm	Promotes the development of flt3-itd mutant white blood	[82]
circAmotl1	Binds PDK1 and AKT1	cytoplasm	Protein scaffolds promote phosphorylation and promote cardiac protective nuclear translocation	[20]
circFOXO3	Binds to the complex of ID-1, CDK2-p21 and p53-MDM2	cytoplasm	Affects cell senescence, cell cycle and apoptosis	[83, 84]
circZNF609	Translation template	cytoplasm	Specifically controls the proliferation of myoblasts	[23]
circSHPRH	Translation template	cytoplasm	Inhibits cell proliferation and tumor suppressors	[96]
circ-LINCPINT	Translation template	cytoplasm	Inhibits cell proliferation and tumor suppressors	[97]
circFBXW7	Translation template	cytoplasm	Inhibits the proliferation of cancer cells and the acceleration of the cell cycle	[98]
circPPP1R12A	Translation template	cytoplasm	Inhibits cell proliferation and tumor suppressors	[21]

### MiRNA sponges

Numerous studies have demonstrated that circRNAs can play a vital regulatory role as miRNA sponges in tumours. The most representative example is ciRS-7, which has more than 70 selectively conserved miRNA targets [17]. CiRS-7 can inhibit the activity of miR-7 and lead to an increased level of the target of miR-7. In addition, the testicular-specific sex-determining region Y circRNA (circ-Sry), can act as a miR-138 sponge. Gao and Ye et al. used microarray analysis to select the most significantly upregulated carcinogenic factor (circ-sox4) in lung adenocarcinoma (LUAD) tissues and found that circ-sox4 promoted the proliferation, invasion, and migration of LUAD cells by sponging miR-1270 and upregulating PLAGL2 [74]. In bladder cancer, circTCF25 acts as a miRNA sponge, suppressing the functions of miR-103a-3p and miR-107 in tumour tissues and increasing the expression of cyclin-dependent kinase 6 (CDK6), leading to tumour cell proliferation [75]. Pan et al. found a carcinogenic circRNA, circ-TFF1, which is produced from the host gene trefoil factor 1 (TFF1) located on chromosome 21q22.3, and found that circ-TFF1 plays a carcinogenic role in breast cancer by regulating the miR-326/TFF1 axis [76]. All of the above results suggest that miRNA sponging by circRNAs is a common phenomenon [17].

Intriguingly, studies have also shown that circRNAs can act as competing endogenous RNAs (ceRNAs) to influence epithelial-mesenchymal transition (EMT) in tumours. EMT can affect the expression patterns of cell adhesion, migration, proliferation, apoptosis, and other genes and can modify cell behaviour to induce drastic changes [77]. First, a study showed that the expression of hundreds of circRNAs is induced during EMT in humans [43]. Then, Wei et al. identified the tumour suppressor circPTPRA through an initial microarray analysis of non-small cell lung cancer (NSCLC) samples and found that circPTPRA inhibited EMT in NSCLC cells by binding to miR-96-5p [78]. The tumour suppressor circRIP2 induces EMT in bladder cancer through competitive binding to miR-1305 and activation of the transforming growth factor-β2 (Tgf-β2)/Smad3 pathway to promote the proliferation, invasion, and migration of bladder tumour cells [79]. However, circAMOTL1L acts as a sponge by binding to miR-193a-5p in prostate cancer (Pca) cells, which alleviates the inhibitory effects of miR-193a-5p on the protocadherin (PCDHA) gene cluster, promotes prostate cancer cell EMT in vivo, and leads to the growth of prostate cancer in vivo [80]. To date, circRNAs have been found to stabilize miRNAs in addition to acting as molecular sponges for miRNAs. Through miRNA expression profiling and analysis of AGO2-CLIP seq data, researchers showed that circCSNK1G3 may function by binding to miR-181b/d. Interestingly, the interaction of circCSNK1G3 with miR-181b/d does not inhibit the activity of miR-181b/d, but the reduction in circCSNK1G3 expression abolishes the ability of miR-181b/d to inhibit the expression of its target genes (CBX7, CDK1, and CDC25A) [49]. This pattern is inconsistent with the usual pattern of circRNA action. However, competitive binding of circRNAs to miRNAs remains the primary mechanism underlying their regulatory functions in tumours. Research on circRNAs can provide potential targets for cancer treatment.

### RBPs

RBPs are a broad class of proteins that interact with RNA molecules and can play key roles in RNA post-transcriptional regulation, tissue development, and diseases [68].

#### CircRNAs bind to RBPs to regulate transcript splicing and parental gene transcription

As a regulatory protein of circRNAs, muscleblind (MBL) in Drosophila can promote the circularization of mRNA precursors. The MBL circRNA (circMb1) and its flanking introns contain conserved sites that bind specifically to MBL. At low concentrations of MBL, the MBL gene produces a linear mRNA transcript, which is translated into the MBL protein. In contrast, at high concentrations of MBL, the MBL protein binds to its precursor RNA, prompting the formation of circMbl from exon 2, preventing the production of additional MBL protein and exerting negative feedback regulation [1]. An enriched CiRNA, ci-ankrd52, accumulates mainly in the nucleus and promotes the transcription of ANKRD52 through cis regulation of RNA Pol II. Knockout of ci-ankrd52 can reduce the expression of its parental gene [32]. Additionally, EIciRNAs, which are composed of exons and introns, promotes transcription. A study showed that EIciRNAs such as circIIF3J and circPAIP2, which are localized mainly in the nucleus, interact with the U1 small nuclear ribonucleoprotein (U1 snRNP) and RNA Pol II to enhance the transcription of their parental genes. Knockout of circEIF3J and circPAIP2 reduced the transcript levels of EIF3J and PAIP2, respectively [81]. Interestingly, both of these EIciRNAs are present in the nucleus and act as cis-regulatory elements to promote the expression of their parental genes, but the other potential functions of these EIciRNAs, such as trans regulation, are unknown.

#### CircRNAs bind to RBPs to regulate translation and act as protein scaffolds

CircRNAs can bind to specific RBPs and regulate the interaction between RBPs and their target RNAs. Sun et al. found that circMYBL2 regulates the mRNA translation efficiency of the oncogene FMS-like tyrosine kinase-3 (FLT3) by recruiting the RBP PTBP1, thus promoting the occurrence and development of white blood cells harbouring FLT3-internal tandem duplication (ITD) mutations [82]. That study was the first to report that circRNAs play a decisive regulatory role in the translation process as RNA-protein complexes [82]. In addition, circRNAs may act as a “scaffold” for RBPs, binding to multiple RBPs and promoting stable interactions through the potentially increased stability of circRNA transcripts. For example, circAmotl1 physically binds to 3-phosphoinositide-dependent protein kinase 1 (PDK1) and protein kinase B (AKT1) to promote PDK1-dependent AKT1 phosphorylation. In addition, circAmotl1 promotes the cardioprotective nuclear translocation of PAKT [20]. CircFOXO3 can affect cell senescence, the cell cycle, and apoptosis by interacting with the anti-ageing 132 protein ID-1, the CDK2-p21 complex, and the p53-MDM2 complex [83, 84]. In summary, we speculate that circRNAs may also function as sequence-targeting elements. Interactions between circRNAs and RBPs can also mediate various biological activities, such as cell proliferation, differentiation, motility, apoptosis, senescence, and the cellular response to oxidative stress, through post-transcriptional regulation [83, 85]. Some studies have shown that specific proteins can synergistically bind to multiple circRNAs in the cytoplasm to produce a molecular repository of proteins that respond rapidly to extracellular stimuli. This process can achieve a rapid immune response after viral infection [18].

### Protein translation

CircRNAs are considered non-coding RNAs because they lack 3′ and 5′ ends [86]. However, in 2015, Abe et al. provided strong evidence that endogenous circRNAs can act as translation templates [87]. In the acellular *Escherichia coli* translation system, circRNAs with an infinite ORF were effectively translated through the roll-ring amplification technique (RCA). These results suggest that circRNAs without poly (A) tail or cap structures can be translated into proteins [87]. Since that discovery, accumulating evidence has shown that circRNAs can encode regulatory proteins/peptides [88] and that these functional proteins/peptides can regulate biological processes and affect tumour occurrence, invasion, and metastasis [89].

#### Translation patterns based on the IRES

Eukaryotic mRNAs are translated through a typical cap-dependent translation mechanism [90]. However, under conditions such as cellular stress exposure or viral infection, mRNA translation can be initiated through a cap-independent alternative translation mechanism via the internal ribosome entry site (IRES) [91]. The IRES can directly recruit ribosomes, perform ribosomal assembly and in-frame protein translation, and initiate protein translation independent of the 5′ cap structure and direct translation [92]. In 2017, Legnini et al. found that circZNF609 in mouse and human muscle cells explicitly controls the proliferation of muscle cells. During myogenesis, heat shock activates circZNF609 translation, and the UTR of circZNF609 can act as an IRES to support protein translation in a splice-dependent and cap-independent manner [23]. Surprisingly, additional studies have demonstrated that through IRES-mediated translation, circRNAs produce peptides that regulate tumour biological functions [93–95]. CircSHPRH [96], circ-LINCPINT [97], circFBXW7 [98], and circPPP1R12A [21] can translate proteins or short peptide chains in glioma by relying on the IRES-mediated translation mechanism. CircRNAs with more than 50 nucleotides (nt) may contain a hexamer similar to an IRES [88], a feature that indicates the universality of the IRES-mediated circRNA translation mechanism.

#### Translation modes based on m^6^A

In addition to the IRES-mediated circRNA translation mechanism, another important cap-independent translation mechanism is mediated by the presence of methylated adenosine residues in the form of m^6^A in the 5’UTR [99]. m^6^A modification is quite common in mRNAs and ncRNAs [100, 101]. Recently, circRNAs were found to contain numerous short sequences with m^6^A sites [102]. Yun et al. found that m^6^A in the 5’UTR promoted cap-independent translation during heat stress through the protective mechanism of YTHDF2 [102]. In addition, this group found that numerous circRNAs are methylated, and hundreds of endogenous translatable circRNAs containing m^6^A sites were identified by sequencing [102]. Collectively, the above findings demonstrate that the m^6^A-mediated translation is typical for circRNAs [103, 104]. The IRES-mediated and m^6^A-mediated translation mechanisms are two primary cap-independent circRNA translation mechanisms. More mechanisms by which circRNAs are translated into proteins remain to be discovered.

## Potential of circRNAs as biomarkers

The early symptoms of most tumours are not obvious, and patients often miss the best opportunity for treatment due to the lack of specific early diagnostic markers. Therefore, identification of accurate biomarkers and therapeutic targets is urgently needed. CircRNAs are potential biomarkers for the early diagnosis, metastasis, prognosis, and drug resistance of tumours due to their stable structure [11], long half-life [52], tumour specificity [16], and ability to be detected in various body fluids [105–107]. Regarding the early diagnosis of tumours, Ren et al. found that hsa_circ_0043265 exhibited low expression in NSCLC tissues and cells and that it could increase the expression of FOXP2 through sponging miR-25-3p, thus inhibiting NSCLC progression. Thus, hsa_circ_0043265 could be used as a biomarker for the early diagnosis of NSCLC [108]. Li et al. found that circMYLK was highly expressed in liver cancer tissues and cell lines and promoted the occurrence and development of liver cancer by regulating the miR-362-3p/Rab23 axis, thus providing a basis for the early diagnosis and treatment of liver cancer [109]. Regarding tumour metastasis, Yang et al. found that the expression of circPTK2 was upregulated in colorectal cancer (CRC) tissues and that the survival rate of colorectal cancer patients with high circPTK2 expression was lower than that of colorectal cancer patients with low circPTK2 expression. CircPTK2 promotes EMT in colorectal cancer cells both in vivo and in vitro by binding to vimentin at Ser38, Ser55, and Ser82. These results suggest that circPTK2 may be a therapeutic target for metastatic colorectal cancer and a promising biomarker for the early diagnosis of metastasis [110]. Regarding tumour prognosis, Guo et al. found that circBFAR expression was upregulated in pancreatic ductal adenocarcinoma (PDAC). CircBFAR expression was positively correlated with the tumour-node-metastasis (TNM) stage and was associated with poor prognosis in PDAC patients. This circRNA enhancing EMT by binding miR-34b-5p and activating the Met/PI3K/Akt signalling pathway, finally promoting the development of PDAC. CircBFAR could be used as a prognostic indicator and therapeutic target for PDAC [111]. In addition, the expression levels hsa_circ_0124055 and hsa_circ_0101622 in tumour tissues and plasma of patients with thyroid cancer are significantly increased, and the overall survival times of patients with a high expression level of either circRNA were shorter than those of patients with a low expression level of either circRNA. These results suggest that both of these circRNAs are helpful biomarkers for the prognosis and diagnosis of thyroid carcinoma and can be used as clinical therapeutic targets [112]. In addition, regarding drug resistance, hsa_circ_0006528 [113], circMTO1 [114], circ_0001546 [115], and circ-LARP4 [116] exhibit abnormal expression levels in drug-resistant cells, suggesting that they could be used as diagnostic markers for drug resistance in tumours.

CircRNAs have become accepted as biomarkers for multi-stage tumours. If circRNA detection methods can be effectively applied in clinical practice, these methods could be used to diagnose tumours in patients as early as possible and avoid patient distress.

## Mechanisms of circRNAs in drug resistance

**Fig. 3:**
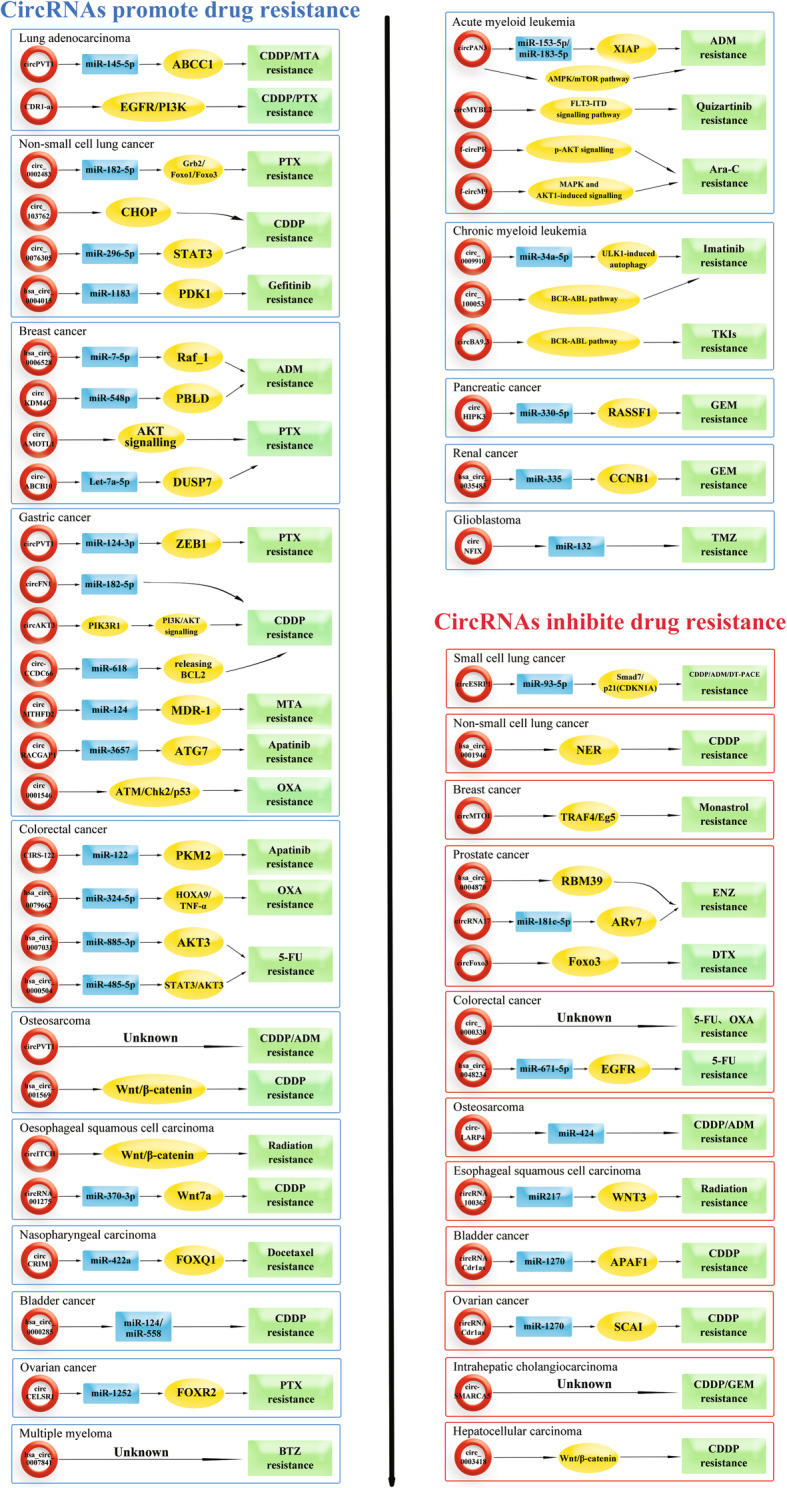
The mechanisms of circRNAs in drug-resistant tumours. Circular RNAs are a double-edged sword in the mechanism of drug-resistant tumors, which can not only promote drug resistance but also inhibit drug resistance


The unique properties and biological functions of circRNAs have indicated their importance in tumorigenesis, tumour growth, metastasis, invasion, drug resistance and radioresistance. Further, these results suggest that circRNAs may become new biomarkers or therapeutic targets for tumours [24–26]. As shown in Fig. 3 and Table 2, we summarize the potential mechanisms of circRNAs in drug-resistant tumours to provide evidence for clinical treatment strategies.

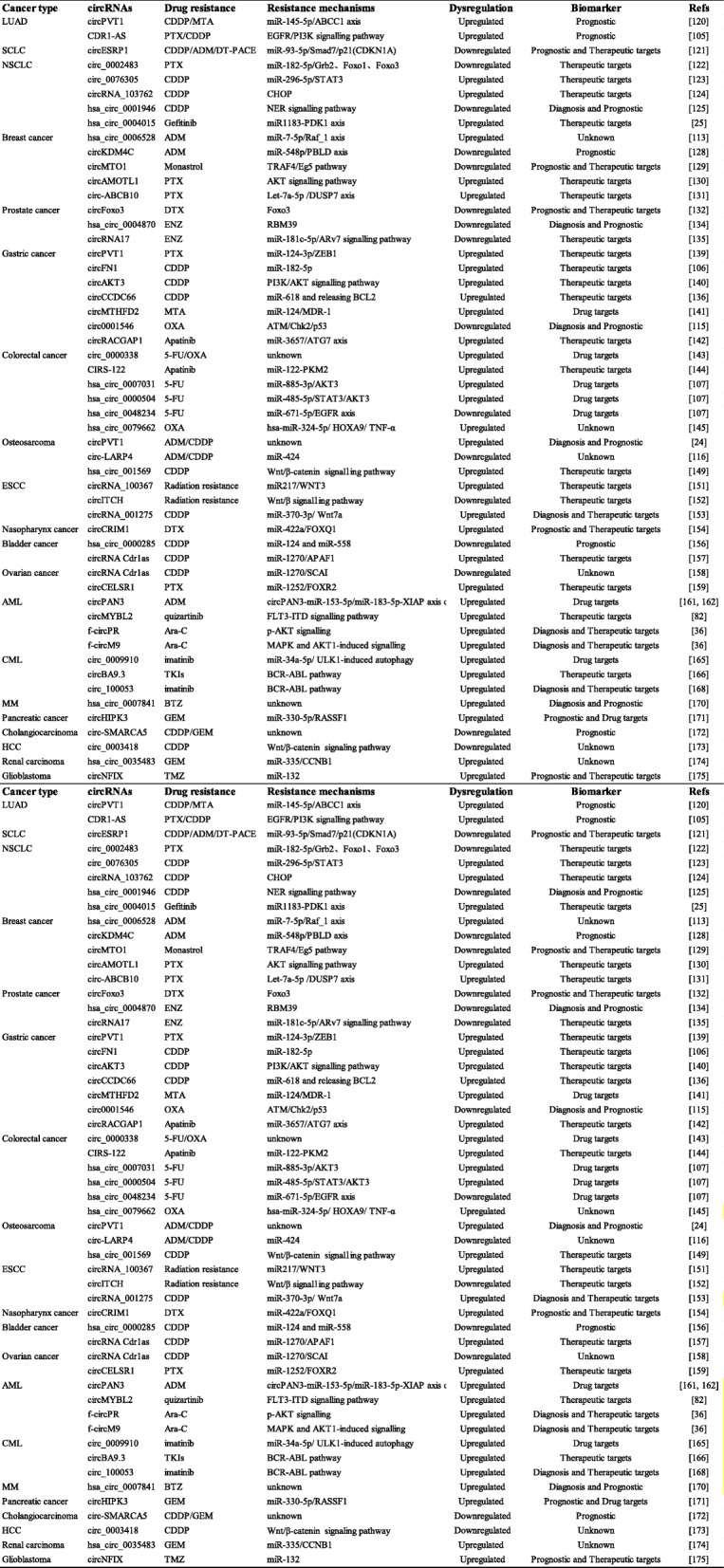

**Table 2:** Overview of circRNAs in drug-resistant tumors

Cancer type	circRNAs	Drug resistance	Resistance mechanisms	Dysregulation	Biomarker	Refs
LUAD	circPVT1	CDDP/MTA	miR-145-5p/ABCC1 axis	Upregulated	Prognostic	[120]
	CDR1-AS	PTX/CDDP	EGFR/PI3K signalling pathway	Upregulated	Prognostic	[105]
SCLC	circESRP1	CDDP/ADM/DT-PACE	miR-93-5p/Smad7/p21(CDKN1A)	Downregulated	Prognostic and Therapeutic targets	[121]
NSCLC	circ_0002483	PTX	miR-182-5p/Grb2、Foxo1、Foxo3	Downregulated	Therapeutic targets	[122]
	circ_0076305	CDDP	miR-296-5p/STAT3	Upregulated	Therapeutic targets	[123]
	circRNA_103762	CDDP	CHOP	Upregulated	Therapeutic targets	[124]
	hsa_circ_0001946	CDDP	NER signalling pathway	Downregulated	Diagnosis and Prognostic	[125]
	hsa_circ_0004015	Gefitinib	miR1183-PDK1 axis	Upregulated	Therapeutic targets	[25]
Breast cancer	hsa_circ_0006528	ADM	miR-7-5p/Raf_1 axis	Upregulated	Unknown	[113]
	circKDM4C	ADM	miR-548p/PBLD axis	Downregulated	Prognostic	[128]
	circMTO1	Monastrol	TRAF4/Eg5 pathway	Downregulated	Prognostic and Therapeutic targets	[129]
	circAMOTL1	PTX	AKT signalling pathway	Upregulated	Therapeutic targets	[130]
	circ-ABCB10	PTX	Let-7a-5p /DUSP7 axis	Upregulated	Therapeutic targets	[131]
Prostate cancer	circFoxo3	DTX	Foxo3	Downregulated	Prognostic and Therapeutic targets	[132]
	hsa_circ_0004870	ENZ	RBM39	Downregulated	Diagnosis and Prognostic	[134]
	circRNA17	ENZ	miR-181c-5p/ARv7 signalling pathway	Downregulated	Therapeutic targets	[135]
Gastric cancer	circPVT1	PTX	miR-124-3p/ZEB1	Upregulated	Therapeutic targets	[139]
	circFN1	CDDP	miR-182-5p	Upregulated	Therapeutic targets	[106]
	circAKT3	CDDP	PI3K/AKT signalling pathway	Upregulated	Therapeutic targets	[140]
	circCCDC66	CDDP	miR-618 and releasing BCL2	Upregulated	Therapeutic targets	[136]
	circMTHFD2	MTA	miR-124/MDR-1	Upregulated	Drug targets	[141]
	circ0001546	OXA	ATM/Chk2/p53	Downregulated	Diagnosis and Prognostic	[115]
	circRACGAP1	Apatinib	miR-3657/ATG7 axis	Upregulated	Therapeutic targets	[142]
Colorectal cancer	circ_0000338	5-FU/OXA	unknown	Upregulated	Drug targets	[143]
	CIRS-122	Apatinib	miR-122-PKM2	Upregulated	Therapeutic targets	[144]
	hsa_circ_0007031	5-FU	miR-885-3p/AKT3	Upregulated	Drug targets	[107]
	hsa_circ_0000504	5-FU	miR-485-5p/STAT3/AKT3	Upregulated	Drug targets	[107]
	hsa_circ_0048234	5-FU	miR-671-5p/EGFR axis	Downregulated	Drug targets	[107]
	hsa_circ_0079662	OXA	hsa-miR-324-5p/ HOXA9/ TNF-α	Upregulated	Unknown	[145]
Osteosarcoma	circPVT1	ADM/CDDP	unknown	Upregulated	Diagnosis and Prognostic	[24]
	circ-LARP4	ADM/CDDP	miR-424	Downregulated	Unknown	[116]
	hsa_circ_001569	CDDP	Wnt/β-catenin signalling pathway	Upregulated	Therapeutic targets	[149]
ESCC	circRNA_100367	Radiation resistance	miR217/WNT3	Upregulated	Therapeutic targets	[151]
	circITCH	Radiation resistance	Wnt/β signalling pathway	Downregulated	Therapeutic targets	[152]
	circRNA_001275	CDDP	miR-370-3p/ Wnt7a	Upregulated	Diagnosis and Therapeutic targets	[153]
Nasopharynx cancer	circCRIM1	DTX	miR-422a/FOXQ1	Upregulated	Prognostic and Therapeutic targets	[154]
Bladder cancer	hsa_circ_0000285	CDDP	miR-124 and miR-558	Downregulated	Prognostic	[156]
	circRNA Cdr1as	CDDP	miR-1270/APAF1	Upregulated	Therapeutic targets	[157]
Ovarian cancer	circRNA Cdr1as	CDDP	miR-1270/SCAI	Downregulated	Unknown	[158]
	circCELSR1	PTX	miR-1252/FOXR2	Upregulated	Therapeutic targets	[159]
AML	circPAN3	ADM	circPAN3-miR-153-5p/miR-183-5p-XIAP axis or AMPK/mTOR pathway	Upregulated	Drug targets	[161, 162]
	circMYBL2	quizartinib	FLT3-ITD signalling pathway	Upregulated	Therapeutic targets	[82]
	f-circPR	Ara-C	p-AKT signalling	Upregulated	Diagnosis and Therapeutic targets	[36]
	f-circM9	Ara-C	MAPK and AKT1-induced signalling	Upregulated	Diagnosis and Therapeutic targets	[36]
CML	circ_0009910	imatinib	miR-34a-5p/ ULK1-induced autophagy	Upregulated	Drug targets	[165]
	circBA9.3	TKIs	BCR-ABL pathway	Upregulated	Therapeutic targets	[166]
	circ_100053	imatinib	BCR-ABL pathway	Upregulated	Diagnosis and Therapeutic targets	[168]
MM	hsa_circ_0007841	BTZ	unknown	Upregulated	Diagnosis and Prognostic	[170]
Pancreatic cancer	circHIPK3	GEM	miR-330-5p/RASSF1	Upregulated	Prognostic and Drug targets	[171]
Cholangiocarcinoma	circ-SMARCA5	CDDP/GEM	unknown	Downregulated	Prognostic	[172]
HCC	circ_0003418	CDDP	Wnt/β-catenin signaling pathway	Downregulated	Unknown	[173]
Renal carcinoma	hsa_circ_0035483	GEM	miR-335/CCNB1	Upregulated	Unknown	[174]
Glioblastoma	circNFIX	TMZ	miR-132	Upregulated	Prognostic and Therapeutic targets	[175]

### Lung cancer

Cisplatin (CDDP), pemetrexed (MTA), paclitaxel (PTX), and gefitinib are effective targeted drugs for lung cancer [117–119]. Numerous experiments have demonstrated that circRNAs play a regulatory role in drug resistance in lung cancers, including LUAD, small cell lung cancer (SCLC), and NSCLC. CircPVT1 is upregulated in LUAD tissues and cell lines with resistance to CDDP and MTA. CircPVT1 was found to mediate CDDP and MTA resistance via the miR-145-5p/ABCC1 axis. CircPVT1 knockout sensitizes tumour cells to CDDP and MTA [120]. Another study showed that upregulation of CDR1-as in LUAD tissues and cell lines is related to PTX and CDDP insensitivity in LUAD patients. CDR1-as promotes chemotherapeutic resistance to PTX and CDDP in LUAD patients through the epidermal growth factor receptor (EGFR)/phosphatidylinositol 3-kinase (PI3K) signalling pathway [105]. In SCLC, circESRP1, which can directly bind to miR-93-5p and upregulate the expression of its downstream target genes Smad7/cyclin-dependent kinase inhibitor 1 (p21), is significantly downregulated in drug-resistant cells. Finally, a negative feedback loop is formed. At the same time, TGF-β-mediated EMT is regulated to enhance the sensitivity to CDDP, adriamycin (ADM), and etoposide (DT-PACE). In addition, both overexpression of circESRP1 and inhibition of the TGF-β signalling pathway can change the tumour response to chemotherapy [121]. Circ_0002483 is downregulated in NSCLC cells and can regulate its target genes growth factor receptor-bound protein2 (Grb2), forkhead box protein O1 (Foxo1), and forkhead box protein O3 (Foxo3) by sponging miR-182-5p, thus enhancing the sensitivity of NSCLC cells to PTX [122]. In another study, circ_0076305 and circRNA_103762 were found to be significantly upregulated in CDDP-resistant NSCLC tissues and cell lines. Circ_0076305 can regulate CDDP resistance in NSCLC cells by binding to miR-296-5p and acting on the target gene STAT3 [123]. CircRNA_103762 promotes CDDP resistance in NSCLC by targeting DNA damage-inducible transcript 3 (CHOP) [124]. Hsa_circ_0001946 is downregulated in NSCLC cells and has been proven to reduce the sensitivity of NSCLC cells to CDDP by regulating the nucleotide excision repair (NER) signalling pathway; promoting the survival, proliferation, migration, and invasion of NSCLC cells; and inhibiting apoptosis [125]. Finally, upregulation of hsa_circ_0004015 in NSCLC cells can enhance the resistance of lung cancer cells to gefitinib via the circRNA/miR1183-PDK1 axis [25].

### Breast cancer

Chemotherapy is an effective method to prevent breast cancer recurrence and metastasis after surgical treatment [126]. However, chemotherapeutic resistance remains a major problem. Hsa_circ_0006528 is upregulated in ADM-resistant breast cancer cells, possibly via the circRNA/miR-7-5p/Raf1 axis [113]. Low expression levels of miR-7 have long been proven to confer resistance to breast cancer chemotherapy [127]. In another study of ADM-resistant breast cancer, circKDM4C downregulation was found to inhibit tumour progression and alleviate ADM resistance by regulating the miR-548p/PBLD axis [128]. Additionally, the expression level of circMTO1 (hsa_circ_007874) in monastrol-resistant breast cancer cell lines is significantly reduced compared with that in monastrol-sensitive breast cancer cell lines, and overexpression of circMTO1 can reverse monastrol resistance through the circRNA/TNF receptor-associated factor 4 (TRAF4)/Eg5 pathway [129]. In addition, Ma et al. found that circMOTL1, which may play an essential role in the PTX resistance of breast cancer cells by regulating the AKT pathway, promoting anti-apoptotic protein expression, and inhibiting pro-apoptotic protein expression, is upregulated in breast cancer [130]. Yang et al. found that the expression of circ-ABCB10 was upregulated in breast cancer cells. Circ-ABCB10 mediates the PTX resistance, apoptosis, invasion and autophagy of breast cancer cells through the let-7a-5p/DUSP7 axis [131].

### Prostate cancer

CircFoxo3 can decrease the survival, migration, invasion, and docetaxel (DTX) resistance of prostate cancer cells and can influence DTX resistance through the circRNA/Foxo3/EMT pathway [132]. Currently, androgen deprivation therapy (ADT) with enzalutamide (ENZ) can prolong the survival of patients with castration-resistant prostate cancer (CRPC). However, most patients develop ENZ resistance [133]. Hsa_circ_0004870 is downregulated in ENZ-resistant cells and plays a key role in mediating ENZ resistance in CRPC cells through RBM39 [134]. Another study showed that circRNA17 may suppress ENZ resistance in ENZ-resistant CRPC tumour cells by altering miR-181c-5p/ARv7 signalling [135].

### Gastric cancer

CDDP, MTA, PTX, and oxaliplatin (OXA) are commonly used in gastric cancer (GC) chemotherapy [136–138]. However, patients always acquire chemotherapeutic resistance after treatment, which limits the overall clinical efficacy of the treatment. CircPVT1 is a carcinogenic factor in GC, mediating PTX resistance by upregulating ZEB1 via miR-124-3p [139]. Huang et al. found that circFN1 can promote CDDP-induced GC cell activity and inhibit GC cell apoptosis in vivo and in vitro. CircFN1 inhibits GC cell apoptosis through sponging miR-182-5p and promotes CDDP resistance in GC, suggesting that circFN1 could be a therapeutic target in GC patients receiving CDDP treatment [106]. As another example of circRNA involvement in CDDP resistance in GC, circAKT3 can regulate phosphoinositide 3-kinase regulatory subunit 1 (PIK3R1), while PIK3R1 increases CDDP resistance by activating the PI3K/AKT signalling pathway [140]. Zhang et al. found that circCCDC66 is an important regulator of CDDP resistance and is highly expressed in CDDP-resistant cells and tissues. In vitro and in vivo experiments showed that circCCDC66 inhibits apoptosis and promotes drug resistance by targeting miR-618 and releasing B cell lymphoma-2 (BCL2) [136]. Via microarray analysis, Xu et al. identified the upregulation of circMTHFD2, which bound directly to miR-124 as a molecular sponge, in GC cells. This binding induced an increase in MDR-1 protein expression, ultimately enhancing MTA resistance in GC cells [141]. Circ_0001546 is upregulated in GC tissues and cells, which can increase ATM expression and inhibit cell proliferation and OXA resistance by activating the ATM/Chk2/p53-dependent pathway [115]. In GC cells, silencing circRACGAP1 inhibits apatinib-induced autophagy, which can be rescued by miR-3657 expression. Knockout of the circRACGAP1 gene endows GC cells with sensitivity to apatinib by inhibiting autophagy. CircRACGAP1 was found to mediate apatinib resistance through the circRACGAP1/miR-3657/ATG7 axis [142].

### Colorectal cancer

Hon et al. found that circ_0000338 has a tumour-suppressive effect in colorectal cancer and can enhance the chemosensitivity of colorectal cancer cells. Knockout of hsa_circ_0000338 in HCT116-R cells increases 5-fluorouracil (5-FU) and OXA resistance and may have a dual regulatory effect. Hsa_circ_0000338 was selectively transfected into HCT_116-P cells cocultured with HCT_116-R exosomes, which exhibited a more robust response to drug therapy than control cells. Hsa_circ_0000338 may play a carcinogenic role in HCT116-R exosomes and enhance the drug resistance of the recipient cells [143]. In addition, CIRS-122 (hsa_circ_0005963) acts as a sponge for miR-122, which targets PKM_2, and is positively correlated with chemotherapeutic resistance. Studies have shown that exosomes from OXA-resistant cells transport CIRS-122 to sensitive cells, thereby promoting glycolysis and chemotherapeutic resistance through upregulation of miR-122 sponging and PKM2 expression. In addition, extracellular transport of si-CIRC-122 inhibits glycolysis and reverses OXA resistance in vivo by regulating the CIRS-122/miR-122/PKM2 pathway [144]. Xiong et al. investigated circRNA regulation in 5-FU-resistant colorectal cancer cells for the first time, finding that the most strongly upregulated circRNAs—hsa_circ_0007031 and hsa_circ_0000504—promoted 5-FU resistance by regulating the circRNA/miR-853-3p/AKT3 and circRNA/miR-485-5p/STAT3/AKT3 signalling pathways or by regulating Bcl2 protein expression. In addition, the authors speculated that downregulation of hsa_circ_0048234, which has four miR-671-5p binding sites, may promote drug resistance by upregulating the miR-671-5p/EGFR axis [107]. Lai et al. found that hsa_circ_0079662, which can bind to hsa-miR-324-5p, regulate the target gene HOXA9, and induce resistance to the chemotherapeutic drug OXA in colorectal cancer via the tumor necrosis factor-α (TNF-α) pathway, is upregulated in drug-resistant colorectal cancer cells [145].

### Osteosarcoma

Osteosarcoma (OS) is one of the most common primary bone tumours. CircPVT1 is significantly upregulated in OS and can reduce the resistance of OS cells to ADM and CDDP by decreasing the expression of the ABCB1 gene, which is related to classical drug resistance, after knockout [24]. However, the detailed mechanism is unclear. Overexpression of circ-LARP4 increases the sensitivity of MG63 cells to CDDP and ADM but does not significantly affect sensitivity to methotrexate (MTX). In addition, overexpression of miR-424 reduces the chemosensitivity of circ-LARP4-overexpressing MG63 cells [116]. In addition, experimental results have shown that circ-LARP4 can affect the development of GC [146], ovarian tumours [147], and oesophageal squamous cell carcinoma (ESCC) [148]. Zhang et al. found that hsa_circ_001569 is upregulated in CDDP-resistant OS cells, which promotes cell proliferation by activating the Wnt/β-catenin pathway and enhances resistance to CDDP [149].

### Oesophageal squamous cell carcinoma

Radiotherapy is a main treatment for patients with ESCC. However, radioresistance is a historical reason for the failure of ESCC therapy and local tumour recurrence [150]. In ESCC, circRNA_100367 attenuates the radioresistance of oesophageal tumour cells via the miR217/Wnt3 pathway. CircRNA_100367 inhibits the proliferation and migration of ESCC cells [151]. In addition, circITCH is downregulated in ESCC tissues. As previous radioresistance studies indicated, circITCH may inhibit the expression of its target gene by inhibiting the Wnt/β-catenin signalling pathway, thus affecting radioresistance in ESCC [152]. CircRNA_001275 is significantly upregulated in CDDP-resistant oesophageal cancer tissues and cells. Overexpression of circRNA_001275 promotes the proliferation and invasion of CDDP-resistant cells, reduces their apoptosis, and promotes CDDP resistance by upregulating Wnt7a expression by sponging miR-370-3p [153].

### Nasopharyngeal carcinoma

In highly metastatic nasopharyngeal carcinoma (NPC) cells, circCRIM1 is upregulated. Overexpression of circCRIM1 promotes NPC cell metastasis and EMT. CircCRIM1 can competitively bind miR-422a and block the inhibitory effect of miR-422a on its target gene FOXQ1, resulting in metastasis, EMT, and DTX resistance in NPC [154]. Additionally, upregulation of circCRIM1 is associated with poor survival of NPC patients. Via the development of a prognostic model based on circCRIM1 expression and N staging, the risk of distant metastasis and the therapeutic response to DTX-induced chemotherapy in NPC patients can be effectively predicted.

### Bladder cancer and ovarian cancer

Bladder cancer is a common tumour of the urinary system. Most (75–80%) bladder cancer patients receive transurethral resection; however, those with advanced bladder cancer can receive only chemotherapy or radiotherapy [155]. However, due to chemotherapeutic resistance, some patients do not benefit from this treatment. Chi et al. studied the differential expression of circRNAs in bladder cancer cell lines and found that the expression of hsa_circ_0000285 in CDDP-resistant cells is almost three times that in CDDP-sensitive cells. The possible mechanism underlying this difference may be related to the expression of miR-124 and miR-558 [156]. Another study showed that circCdr1as sensitizes bladder cancer cells to CDDP by restoring the expression of APAF1, which is decreased by miR-1270. APAF1 silencing decreased the sensitivity of bladder cancer cells to CDDP [157]. In addition, in ovarian cancer, circCdr1as reduces CDDP resistance by inhibiting miR-1270 and upregulating SCAI. Similarly, overexpression of Cdr1as inhibits the proliferation of ovarian cancer cells and promotes CDDP-induced apoptosis [158]. CircRNAs are currently considered essential regulators of tumour development and progression. Zhang et al., through gene chip analysis, found that circCELSR1 (hsa_circ_0063809) is upregulated in PTX-resistant ovarian cancer tissues and cells. CircCELSR1 regulates the expression of FOXR2 via miR-1252 and mediates the resistance of ovarian cancer cells to PTX [159]. Silencing of circCELSR1 enhances the cytotoxic effect of PTX in ovarian cancer cells.

### Acute myeloid leukaemia

Acute myeloid leukaemia (AML) is a highly heterogeneous haematologic malignancy. Drug resistance and recurrence are the key factors in the failure of leukaemia treatment [160]. Shang et al. found that circPAN3, which may be a key regulatory factor for acquired chemoresistance in AML, is upregulated in drug-resistant AML cells and mediates ADM resistance through different pathways. Autophagy can be regulated through the circPAN3-miR-153-5p/miR-183-5p-XIAP axis or the AMPK/mTOR pathway, which act as autophagy inducers, to promote ADM resistance in AML cells [161, 162]. Sun et al. found that circMYBL2 expression is higher in AML patients with FLT3-ITD mutations than in AML patients without FLT3-ITD mutations. Knockout of the circMYBL2 gene specifically inhibits the proliferation of FLT3-ITD+ AML cells and overcomes acquired resistance to quizartinib [82]. In addition, Guarnerio et al. discovered a new class of circRNAs, f-circRNAs [36]. This type of circRNA was later identified in the MiOncoCirc database [37]. F-circRNAs cannot trigger tumorigenesis alone, but in combination with other carcinogenic stimuli, they can promote the development of leukaemia and acquired drug resistance. Expression of both f-circPR and f-circM9 leads to an increase in cell proliferation and transformation, but f-circM9 can trigger both mitogen-activated protein kinase (MAPK) and AKT1 signalling, thereby affecting drug resistance. In the presence of f-circPR, the p-AKT level is increased only slightly but affects drug resistance [36].

### Chronic myeloid leukaemia

Chronic myeloid leukaemia (CML) is a disorder of uncontrolled myeloid stem cell proliferation characterized by the Philadelphia chromosome [163]. The development of imatinib and other tyrosine kinase inhibitors (TKIs) has altered the course of the disease; however, resistance develops in approximately 13% of patients [164]. Circ_0009910, which can regulate ULK1-induced autophagy by targeting miR-34a-5p and accelerate the development of imatinib resistance in CML cells, is upregulated in the serum and cells of imatinib-resistant CML patients [165]. In addition, circBA9.3, a circRNA derived from BCR-ABL1, can promote cell proliferation by upregulating the protein expression levels of c-ABL1 and BCR-ABL1 and make CML cells resistant to TKIs, including imatinib, nilotinib, and dasatinib [166]. Ping et al. found that the expression of circ_100053 in imatinib-resistant CML patients is higher than that in imatinib-sensitive patients. Mutations in the BCR-ABL kinase domain (KD) often lead to imatinib resistance [167]. Thus, circ_100053 may regulate imatinib resistance by regulating the BCR-ABL pathway [168].

### Multiple myeloma

Multiple myeloma (MM) is a haematologic malignancy caused by abnormal proliferation of bone marrow plasma cells. Although new treatments have greatly improved the prognosis of MM, its incidence has been increasing annually. A main factor contributing to this phenomenon is the high heterogeneity of MM cells, which leads to disease recurrence and drug resistance in patients [169]. Gao et al. found that the expression of hsa_circ_0007841 is significantly upregulated in MM cell lines and bortezomib (BTZ)-resistant cell lines and that the hsa_circ_0007841 expression level is significantly higher in MM patients with BTZ resistance than in MM patients with BTZ sensitivity. Therefore, upregulation of hsa_circ_0007841 may be involved in BTZ tolerance in MM patients [170].

### Other cancers

CircRNAs also play an essential role in drug resistance in other tumours. CircHIPK3 is upregulated in gemcitabine (GEM)-resistant pancreatic cancer cells. Experiments proved that circHIPK3 targets RASSF1 through miR-330-5p, promotes GEM resistance in pancreatic cancer cells, and regulates cell proliferation, invasion, migration, EMT, and apoptosis [171]. In addition, in cholangiocarcinoma, downregulation of circ_SMARCA5 inhibits the proliferation of CDDP- and GEM-treated cells, reduces the relative cell survival rate, and reduces the inhibitory concentration (IC) of CDDP and GEM by 50%. Circ_SMARCA5 has potential application value in monitoring disease progression and predicting prognosis in intrahepatic cholangiocarcinoma (ICC) [172]. Circ_0003418 is downregulated in hepatocellular carcinoma (HCC) tissues and cell lines and is associated with the tumour size, TNM stage, and HBsAg level. Inhibition of circ_0003418 enhances the CDDP resistance of HCC cells in vivo and in vitro. Circ_0003418 gene knockout activates the Wnt/β-catenin signalling pathway in HCC cells. However, after inhibition of the Wnt/β-catenin pathway, the effect of circ-0003418 on the CDDP sensitivity of hepatoma cells was reversed [173]. In renal cancer, hsa_circ_0035483 promotes autophagy and tumour growth by regulating the miR-335/CCNB1 axis and enhances GEM resistance, and silencing hsa_circ_0035483 can enhance GEM sensitivity [174]. Another study showed that exosomal CircNFIX is upregulated in the serum of temozolomide (TMZ)-resistant patients. CircNFIX can interact directly with miR-132 and enhance the TMZ sensitivity of drug-resistant glioma cells as well as promote cell migration and invasion and inhibit apoptosis [175].

## Conclusions

With the development of modern medicine, tumour treatment is progressing from a traditional treatment model to a targeted treatment model. Targeted therapy can specifically kill tumour cells without affecting the normal peritumoral cells. To date, numerous drugs have been developed for targeted therapy. For example, targeted drugs for GC include PTX, CDDP, MTA, OXA, and apatinib; targeted drugs for colorectal cancer include 5-FU and OXA; and targeted drugs for pancreatic tumours include GEM. However, clinically, tumour cells gradually develop resistance to targeted drugs.

CircRNAs are widely distributed in eukaryotic cells and have a long half-life. Moreover, the expression levels of circRNAs are tissue- and developmental stage-specific. Therefore, we believe that circRNAs have potential as tumour markers and therapeutic targets. However, we found that circRNAs act as a double-edged sword in chemoresistance, not only promoting but also suppressing drug resistance. CircPVT1 promotes CDDP and MTA resistance in LUAD by targeting miR-145-5p and increasing the chemosensitivity of tumour cells. In contrast, monastrol resistance can be reversed in breast cancer via the TRAF4/Eg5 pathway through overexpression of circMTO1. CircRNAs also have a dual regulatory effect in some tumour chemoresistance mechanisms. For example, hsa_circ_0000338 in tumour cells can inhibit tumour growth, but exosomal hsa_circ_0000338 has a carcinogenic effect.

CircRNAs play an essential role in chemoresistance, but the mechanism is not entirely clear. Hidden mechanisms of resistance will lead us to recognize the importance of circRNAs in human tumours. With continuous improvements in circRNA databases and detection technology, we believe that circRNAs will be applied clinically and provide a new approach for tumour treatment.

## Data Availability

Not applicable.

## Abbreviations

CircRNAs: Circular RNAs
ncRNA: Non-coding RNA
RNase R: Ribonuclease R
miRNAs: microRNAs
RBPs: RNA-binding proteins
lncRNA: Long non-coding RNA
EciRNAs: Exon circRNAs
ElciRNAs: Exon-intron circRNAs
CiRNAs: Intronic circRNAs
pre-mRNA: Premessenger RNA
f-circRNAs: Fusion-circRNAs
rt-circRNAs: Read-through circRNAs
UTR: Untranslated Regions
MIR: Mammalian-Wide Interspersed Repeats
m^6^A: N^6^-methyladinosinek
ORF: Open Reading Frame
RIP: RNA Binding Protein Immunoprecipitation
QKI: Quaking
FUS: Fused in sarcoma
SRSF3: Serine/arginine-rich splicing factor 3
hnRNPs: Heterogeneous nuclear ribonucleoproteins
DHX9: ATP-dependent RNA helicase A
ILF3: Interleukin Enhancer Binding Factor 3
AGO2: Argonaute 2
RNAi: RNA interference
YTHDF2: YTH domain-containing family protein 2
HRSP12: Heat- responsive protein 12
RNase P: Ribonuclease P
PKR: Double-stranded RNA-activated protein kinase
RNase L: Ribonuclease L
SRD: Structure-Mediated RNA Decay
YTHDC1: YTH domain-containing family protein 1
FISH: Fluorescence in situ hybridization
LUAD: Lung adenocarcinoma
CDK6: Cyclin-dependent kinase 6
TFF1: Trefoil factor 1
ceRNAs: Competing endogenous RNAs
EMT: Epithelial mesenchymal transition
NSCLC: Non-small-cell lung cancer
Tgf-β2: Transforming growth factor-β2
PCa: Prostatic tumor
PDAC: Pancreatic ductal adenocarcinoma
MBL: Muscle-blind
RNA Pol II: RNA polymerase II
U1 snRNP: U1 small nuclear ribonucleoprotein
PDK1: 3-phosphoinositide- dependent protein kinase 1
IRES: Internal ribosome entry site
PDAC: Pancreatic ductal adenocarcinoma
RCA: Roll ring amplification
CDDP: Cisplatin
MTA: Pemetrexed
PTX: Paclitaxel
SCLC: Small cell lung cancer
p21: Cyclin-dependent kinase inhibitor 1
EGFR: Epidermal Growth Factor Receptor
PI3K: Phosphatidylinositol 3-kinase
Grb2: Growth factor receptor-bound protein2;Foxo1 forkheadboxclass1
Foxo 3: Forkheadboxclass 3
ADM: Adriamycin
TRAF4: TNF receptor-associated factor 4
DTX: Docetaxel
ADT: Androgen deprivation therapy
CRPC: Castration resistant prostate cancer
ENZ: Enzalutamide
OXA: Oxaliplatin
GC: Gastric cancer
PIK3R1: Phosphoinositide-3-Kinase Regulatory Subunit 1
BCL2: B cell lymphoma-2
CRC: Colorectal cancer
5-FU: 5-fluorocrail
TNF-α: Tumor necrosis factor-α
OS: Osteosarcoma
MTX: Methotrexate
ESCC: Esophageal squamous cell carcinoma
NPC: Nasopharyngeal carcinoma
AML: Acute myeloid leukemia
FLT3: FMS-like tyrosine kinase-3
ITD: tandem repeat
MAPK: Mitogen-activated protein kinase
CML: Chronic myeloid leukemia
TKIs: Tyrosine kinase inhibitors
KD: Kinase domain
MM: Multiple myeloma
BTZ: Bortezomib
GEM: Gemcitabine
ICC: Intrahepatic cholangiocarcinoma
TNM: Tumor Node Metastasis
TMZ: Temozolomide
DT-PACE: Etoposide
